# Supplementary material for: Chiral Separation by Flows: The Role of Flow Symmetry and Dimensionality
Source: Sci Rep. 2016 Oct 14;6:35144. doi: 10.1038/srep35144 (PMC5064324; doi:10.1038/srep35144)
Supplement: Supplementary Information [file srep35144-s1.pdf]

Chiral Separation by Flows: The Role of Flow Symmetry and Dimensionality

Sunghan Roh,[1] Juyeon Yi,[2] and Yong Woon Kim[1]

[1] *Graduate School of Nanoscience and Technology, Korea Advanced Institute of Science and Technology, Deajeon 34141, Korea*

[2] *Department of Physics, Pusan National University, Busan 46241, Korea*

I. PARITY INVERSION

I. 1 Parity inversion of mobility and resistance tensors

Here we present the properties of tensors under parity inversion ($\mathbb{P}$). Consider the equation of motion of an object in the low Reynolds number regime (see chapter 5.2 of Ref. [S1]):

$$\begin{pmatrix} \vec{F} \\ \vec{\mathcal{T}} \end{pmatrix} = \begin{pmatrix} \boldsymbol{\zeta}^{\rm tt} & \boldsymbol{\zeta}^{\rm tr} & \boldsymbol{\zeta}^{\rm te} \\ \boldsymbol{\zeta}^{\rm rt} & \boldsymbol{\zeta}^{\rm rr} & \boldsymbol{\zeta}^{\rm re} \end{pmatrix} \begin{pmatrix} \vec{U} - \vec{v} \\ \vec{\Omega} - \vec{\omega} \\ \mathbf{E} \end{pmatrix} + \begin{pmatrix} \vec{F}^{\rm e} \\ \vec{\mathcal{T}}^{\rm e} \end{pmatrix}, \tag{S1}$$

where $\vec{F}$ and $\vec{\mathcal{T}}$ are net force and torque, $\boldsymbol{\zeta}$'s are the resistance tensors, $\vec{F}^{\rm e}$ and $\vec{\mathcal{T}}^{\rm e}$ are an external force and torque, and the definitions of $\vec{U}$, $\vec{\Omega}$, and $\mathbf{E}$ are given below Eq. (1) in the main text. Note that Eq. (1) is rewriting of Eq. (S1) with $\vec{F} = \vec{\mathcal{T}} = 0$ in the absence of external force and torque for overdamped motions at low Reynolds number regime, where the mobility tensors $\boldsymbol{\mu}$'s are given by

$$\begin{pmatrix} \boldsymbol{\mu}^{\rm tt} & \boldsymbol{\mu}^{\rm tr} \\ \boldsymbol{\mu}^{\rm rt} & \boldsymbol{\mu}^{\rm rr} \end{pmatrix} = \begin{pmatrix} \boldsymbol{\zeta}^{\rm tt} & \boldsymbol{\zeta}^{\rm tr} \\ \boldsymbol{\zeta}^{\rm rt} & \boldsymbol{\zeta}^{\rm rr} \end{pmatrix}^{-1} . \tag{S2}$$

Applying $\mathbb{P}$ operation to Eq. (S1), we get

$$\begin{aligned} \mathbb{P}\begin{pmatrix} \vec{F} \\ \vec{\mathcal{T}} \end{pmatrix} &= \begin{pmatrix} -\vec{F} \\ \vec{\mathcal{T}} \end{pmatrix} \\ &= \left[\mathbb{P}\begin{pmatrix} \boldsymbol{\zeta}^{\rm tt} & \boldsymbol{\zeta}^{\rm tr} & \boldsymbol{\zeta}^{\rm te} \\ \boldsymbol{\zeta}^{\rm rt} & \boldsymbol{\zeta}^{\rm rr} & \boldsymbol{\zeta}^{\rm re} \end{pmatrix}\right] \begin{pmatrix} -\vec{U} + \vec{v} \\ \vec{\Omega} - \vec{\omega} \\ \mathbf{E} \end{pmatrix} + \begin{pmatrix} -\vec{F}^{\rm e} \\ \vec{\mathcal{T}}^{\rm e} \end{pmatrix}, \end{aligned}$$

and therefore,

$$\begin{pmatrix} \vec{F} \\ \vec{\mathcal{T}} \end{pmatrix} = \left[\mathbb{P}\begin{pmatrix} \boldsymbol{\zeta}^{\rm tt} & -\boldsymbol{\zeta}^{\rm tr} & -\boldsymbol{\zeta}^{\rm te} \\ -\boldsymbol{\zeta}^{\rm rt} & \boldsymbol{\zeta}^{\rm rr} & \boldsymbol{\zeta}^{\rm re} \end{pmatrix}\right] \begin{pmatrix} \vec{U} - \vec{v} \\ \vec{\Omega} - \vec{\omega} \\ \mathbf{E} \end{pmatrix} + \begin{pmatrix} \vec{F}^{\rm e} \\ \vec{\mathcal{T}}^{\rm e} \end{pmatrix} .$$

Comparing these equations with Eq. (S1), we find,

$$\begin{aligned} \mathbb{P}\boldsymbol{\zeta}^{\rm tt} &= \boldsymbol{\zeta}^{\rm tt}, \;\; \mathbb{P}\boldsymbol{\zeta}^{\rm tr} = -\boldsymbol{\zeta}^{\rm tr}, \;\; \mathbb{P}\boldsymbol{\zeta}^{\rm rt} = -\boldsymbol{\zeta}^{\rm rt}, \\ \mathbb{P}\boldsymbol{\zeta}^{\rm rr} &= \boldsymbol{\zeta}^{\rm rr}, \;\; \mathbb{P}\boldsymbol{\zeta}^{\rm te} = -\boldsymbol{\zeta}^{\rm te}, \;\; \mathbb{P}\boldsymbol{\zeta}^{\rm re} = \boldsymbol{\zeta}^{\rm re} . \end{aligned} \tag{S3}$$

The mobility tensors, $\boldsymbol{\mu}$'s, under parity inversion can be obtained from the block matrix inversion, Eq. (S2):

$$\begin{aligned} \mathbb{P}\boldsymbol{\mu}^{\rm tt} &= \boldsymbol{\mu}^{\rm tt}, \;\; \mathbb{P}\boldsymbol{\mu}^{\rm tr} = -\boldsymbol{\mu}^{\rm tr}, \\ \mathbb{P}\boldsymbol{\mu}^{\rm rt} &= -\boldsymbol{\mu}^{\rm rt}, \;\; \mathbb{P}\boldsymbol{\mu}^{\rm rr} = \boldsymbol{\mu}^{\rm rr} . \end{aligned} \tag{S4}$$

I. 2 Parity inversion of orientation distribution

In obtaining Eq. (4) in the main text, we use a relation, $\Phi^R(\hat{\varphi}') = \Phi^L(\hat{\varphi})$, without proof. Here, we prove that the relation holds for any arbitrary time t, provided that the initial orientation distributions are identical for left-handed

and right-handed objects. Let us begin with the Fokker-Planck equation (Eq. (26) of Ref. [S2] is written in our notation),

$$\begin{aligned} \partial_t P(\vec{r},\hat{\varphi},t) &= -\nabla_i\left[(U_i+v_{\mathbf{E},i})P\right] - \tilde{\nabla}_i\left[(\Omega_i+\omega_{\mathbf{E},i})P\right] \\ &+ D^{\rm tt}_{ij}\nabla_i\nabla_j P + D^{\rm tr}_{ij}\nabla_i\tilde{\nabla}_j P \\ &+ \tilde{\nabla}_i\left(D^{\rm rr}_{ij}\tilde{\nabla}_j P\right) + \tilde{\nabla}_i\left(D^{\rm rt}_{ij}\nabla_j P\right) . \end{aligned} \tag{S5}$$

Here $D^{mn}_{ij} = 2k_BT\mu^{mn}_{ij}$ and, $\vec{\omega}_{\mathbf{E}} = \boldsymbol{\mu}^{\rm rt}(\boldsymbol{\zeta}^{\rm te} : \mathbf{E}) + \boldsymbol{\mu}^{\rm rr}(\boldsymbol{\zeta}^{\rm re} : \mathbf{E})$. The differential operator with the tilde symbol $\tilde{\nabla}$ represents the orientation differential operator, which is invariant under parity inversion, i.e., $\mathbb{P}\tilde{\nabla} = \tilde{\nabla}$. The Fokker-Planck equation in parity inverted system can be found by using Eqs. (S3), (S4), and $\mathbb{P}\nabla = -\nabla$:

$$\begin{aligned} \mathbb{P}\partial_t P(\vec{r},\hat{\varphi},t) &= \partial_t P'(\vec{r}',\hat{\varphi}',t) \\ &= \nabla_i\left[-(U_i+v_{\mathbf{E},i})P'\right] - \tilde{\nabla}_i\left[(\Omega_i+\omega_{\mathbf{E},i})P'\right] \\ &+ D^{\rm tt}_{ij}\nabla_i\nabla_j P' + (-D^{\rm tr}_{ij})(-\nabla_i)\tilde{\nabla}_j P' \\ &+ \tilde{\nabla}_i\left(D^{\rm rr}_{ij}\tilde{\nabla}_j P'\right) + \tilde{\nabla}_i\left[(-D^{\rm rt}_{ij})(-\nabla_j)P'\right] , \end{aligned}$$

from which one can find that the Fokker Planck equation is invariant under the parity inversion. Therefore, $P(\vec{r},\hat{\varphi},t)$ and $P'(\vec{r}',\hat{\varphi}',t)$ should be identical to each other for an initial condition, $P(\vec{r},\hat{\varphi},t=0) = P'(\vec{r}',\hat{\varphi}',t=0)$, and as a consequence, the distribution function of the orientation should be symmetric as,

$$\begin{aligned} \Phi(\hat{\varphi},t) &= \int d\vec{r} P(\vec{r},\hat{\varphi},t) \\ &= \int d\vec{r}' P(\vec{r}',\hat{\varphi}',t) = \Phi'(\hat{\varphi}',t) . \end{aligned}$$

If considering $\Phi(\hat{\varphi},t)$ as the orientation distribution of left-handed objects, say, $\Phi^L(\hat{\varphi})$, this symmetry property proves that its parity inverted counterpart, $\Phi'(\hat{\varphi}',t)$, corresponding to the orientation distribution of right-handed objects, $\Phi^R(\hat{\varphi}')$ is identical with $\Phi^L(\hat{\varphi})$ at any time.

II. SEPARATION CONDITIONS

II. 1 Non-diagonalizable Jacobian matrix

If the Jacobian matrix $\mathbf{J}$ of an incompressible flow field is defective, it has two degenerate eigenvalues and a distinct eigenvalue. We set $\lambda_1 = \lambda_2$ and $\lambda_3 = -\lambda_1 - \lambda_2$, with $\mathbf{J}$ being traceless for incompressible fluids. For this case, eigenvectors do not span three dimensional space and cannot be employed as basis vectors. Instead, we use a set of vectors $\{\hat{\psi}_1, \hat{\psi}_2, \hat{\psi}_3\}$, which transforms the Jacobian matrix into the Jordan form:

$$\mathbf{J}' = \begin{pmatrix} \lambda_1 & J_I & 0 \\ 0 & \lambda_2 & 0 \\ 0 & 0 & \lambda_3 \end{pmatrix} \tag{S6}$$

with J_I being a unit of the Jacobian matrix component. The equation of motion corresponds to Eq. (7) in the main text is given as

$$\dot{r}_i = \lambda_i r_i + v_{\mathbf{E},i} + \xi^{\rm t}_i + \delta_{i1} J_I r_2 ,$$

where the chirality index is omitted, and the subscript i denotes the component along the basis vectors $\hat{\psi}_i$'s which are not eigenvectors. The formal solution of the equation of motion is given by

$$r_i(t) = e^{\lambda_i t} r_i(0) + X_{\mathbf{E},i}(t) + \Xi_i(t) + \delta_{i1} X^{(\alpha)}_I(t), \tag{S7}$$

where $X_I(t) = J_I \int_0^t dt'\ e^{\lambda_1(t-t')} r_2(t')$. For $i = 2, 3$, this equation is identical to Eq. (8) in the main text for a diagonalizable Jacobian. We examine here the behavior of average displacement and dispersion only for $i = 1$. Taking average for thermal noises and initial positions, we obtain the average displacement

$$\langle r_1(t)\rangle = \langle X_{\mathbf{E},1}(t)\rangle + \int_0^t dt'\ e^{\lambda_1(t-t')}\langle X_{\mathbf{E},2}(t')\rangle .$$

Each component of the drift velocity is bounded as $v_{\mathbf{E},i} \le v_{m,i}$ where $i = 1$ and 2 (see the discussion above Eq. (11)), and we calculate the maximum value of the mean separation, $d(t)$, by using equation for $\langle r_1(t)\rangle$ with letting $v_{\mathbf{E},i} = v_{m,i}$ as

$$\begin{aligned} d(t) &= (v_{m,1}/\lambda_1)\left(e^{\lambda_1 t}-1\right) \\ &+ (J_I v_{m,2}/\lambda_1)\left[te^{\lambda_1 t}+\frac{1}{\lambda_1}\left(1-e^{\lambda_1 t}\right)\right] , \end{aligned} \tag{S8}$$

where $\lambda_1 = \lambda_2$ is used. Meanwhile, the dispersion of the object displacement $\Sigma^2(t)$ can be evaluated as

$$\Sigma^2(t) = \sigma^2(t) + \left\langle (X_{\mathbf{E},1}(t)+X_I(t))^2\right\rangle - \langle X_{\mathbf{E},1}(t)+X_I(t)\rangle^2 \tag{S9}$$

$$\sigma^2(t) = \sigma_0^2 e^{2\lambda_1 t} + \frac{D_{11}}{\lambda_1}\left(e^{2\lambda_1 t}-1\right) + J_I e^{2\lambda_1 t}[2A_1(t)+J_I A_2(t)]. \tag{S10}$$

Here the time dependent functions A_i's are given as

$$\begin{aligned} A_1(t) &= \sigma_0^2 t\cos\vartheta_{12} + \frac{D_{12}}{\lambda_1}\left[t+\frac{1}{2\lambda_1}\left(e^{-2\lambda_1 t}-1\right)\right], \\ A_2(t) &= \sigma_0^2 t^2 + \frac{D_{22}}{2\lambda_1}\left[2t^2-\frac{2}{\lambda_1}t-\frac{1}{\lambda_1^2}\left(-1+e^{-2\lambda_1 t}\right)\right] \end{aligned}$$

with ϑ_{12} being the angle between $\hat{\psi}_2\times\hat{\psi}_3$ and $\hat{\psi}_3\times\hat{\psi}_1$, $D_{ij} = k_B T \boldsymbol{\mu}_{ij}^{\mathrm{tt}}$ is a component of translational diffusion matrix.

Applying similar arguments presented between Eqs. (10) and (14) in the main text, we find that $d(t)/\sigma(t) \gg 1$ and $d(t)/\sigma_0 \gg 1$ is required for an efficient separation. This leads to a condition for λ_1 as

$$\lambda_1^{-1} \gg \max\left[t_D, t_\sigma, \theta(-\lambda)\sqrt{t_\sigma t_J}\right], \tag{S11}$$

where $t_J \equiv 1/J$ is time scale of an object to travel a distance of σ_0 by $J\sigma_0$, which is the flow velocity caused by J at an object placed σ_0 away from the origin. A caution should be mentioned for criterion Eq. (S11): If λ_1 becomes zero, chiral separation does not occur neither along $\hat{\psi}_1$ nor $\hat{\psi}_2$, as the flow field has mirror symmetry about the plane spanned by $\hat{\psi}_1$ and $\hat{\psi}_2$. According to our symmetry argument, average drift velocities parallel to the mirror symmetry plane vanish, and separation is unattainable along the directions parallel to the plane. Meanwhile, object motion along $\hat{\psi}_3$ is governed by the equation of motion identical to Eq. (7) in the main text with a finite drift velocity. The separation condition for $\hat{\psi}_3$ direction is similar to the case of diagonalizable $\mathbf{J}$, and it is always satisfied if $\lambda_1 = 0$.

We further note that there exists the other form of singular defective matrix:

$$\mathbf{J}_2 = \begin{pmatrix} 0 & J_{I_1} & 0 \\ 0 & 0 & J_{I_2} \\ 0 & 0 & 0\,, \end{pmatrix} . \tag{S12}$$

for which the equation of motion is given by

$$\dot{r}_i = v_{\mathbf{E},i} + \xi_i^{\mathrm{t}}(t) + \delta_{i1}J_{I_1}r_2(t) + \delta_{i2}J_{I_2}r_3(t) \ . \tag{S13}$$

The motion expressed by Eq. (S13) is analyzed in a similar way to the previous cases. Without showing the details, we mention that chiral separation can always be attained if $v_{E,i}$ is finite.

II.2 Estimate of an upper bound of c

The separation condition (14) is always fulfilled for two-dimensional flows having $\lambda = 0$. On the other hand, a certain flow pattern, not exactly two dimensional, can still induce an efficient separation. Here we provide a quantitative guide on quasi-two-dimensionality bounded by the largest possible value of c. The quantity c of course relies on the specific features of object shape and orientation, and in order to obtain its exact value, it is necessary to analytically calculate the hydrodynamic mobility and resistance tensors of particles of arbitrary shapes. This task is almost impossible to be done and poses tremendous difficulty. We choose an alternative way resorting to an approximation scheme inspired by previous studies [S3–S7], as explained below.

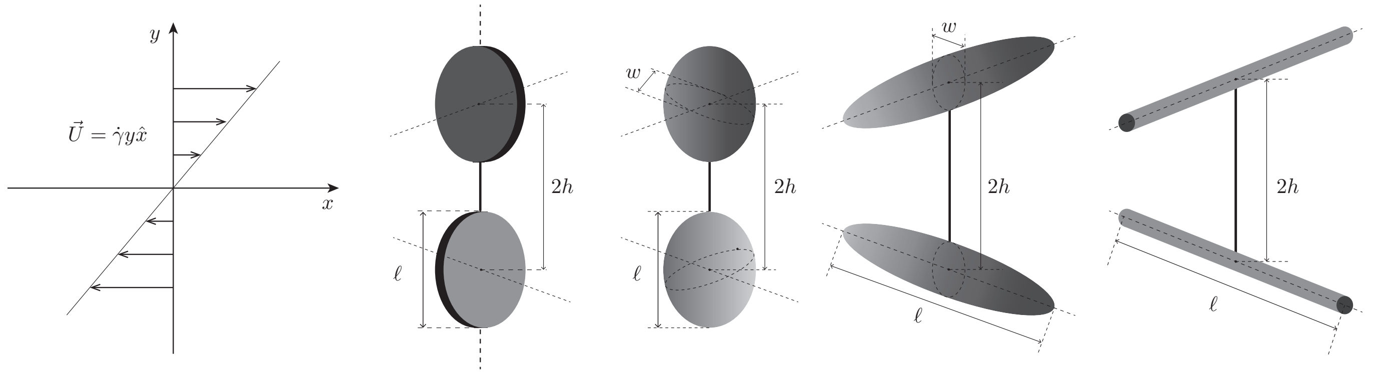

FIG. S1: Shear flow and a skewed propeller consisting of two oblate/prolate spheriods. The lengths of semi-axes are given as $\ell/2$ and $w/2$, respectively. The two spheriods are joined together, by a rigid rod of negligible hydrodynamic resistance, with symmetric axes (dotted lines through the center) orthogonal each other, and the distance between the centers is $2h$. Due to anisotropic hydrodynamic resistances, the drifts of spheroids occur in the $\pm z$ direction. In the limit of the aspect ratio $\ell/w \to \infty$, the prolate (oblate) spheroid resembles a rod (disk).

Since the pioneering works of Kirkwood-Riseman [S3] and Bloomfield [S4], a rigid particle of arbitrarily complex shape has been modeled as an assembly of subunits of simple shape such as spheres (later extended to non-spherical ellipsoids [S7]) for the numerical calculations of the hydrodynamic properties. An arbitrary-shaped rigid object is accordingly thought in our work to be decomposed into elements of ellipsoids like prolate and oblate; they thus cover various simple shapes such as sphere, rod and disk, upon varying aspect ratios. For an analytic calculation of the drift velocity, we make an assumption that the drift velocity of a rigid object in a certain direction cannot be much larger than the maximum of drift velocities that each subunit experiences along that direction in a given flow.

For an order-of-magnitude estimate of possible maximum value of c, the drift velocity of an object is replaced by the drift velocity of a skewed propeller (see Fig. S1) which is a minimal building block with two subunits combined. It has two spheroids arranged in the skew-symmetric way where the symmetric axes are orthogonal to each other. The lengths of semi-axes are $\ell/2$ and $w/2$, and the aspect ratio is defined as ℓ/w. The shape parameters and the orientation of the subunit should be systematically varied to find the largest possible value of c.

We assume that the object is placed in shear flow $\vec{U}(\vec{r}) = \dot\gamma y\hat{x}$, with the rod midpoint located at $\vec{r} = 0$. It is obvious that under a shear flow, the drift velocity would be maximum when the orientation of the skewed propeller is along the y direction. Neglecting hydrodynamic interactions between the spheroids, one can obtain analytic expressions of the translation, rotation, and coupling resistance tensors of the object, as given in Ch. 3 of Ref. [S1]. Then, it is straightforward to obtain the drift velocity given by $\vec{v}_E = \boldsymbol{\mu}^{\rm tt}\left(\boldsymbol{\zeta}^{\rm te} : \mathbf{E}\right) + \boldsymbol{\mu}^{\rm tr}\left(\boldsymbol{\zeta}^{\rm re} : \mathbf{E}\right)$ (see Eq. (2) in the main text). In order to estimate the possible maximum value of c, we consider the value of distance between the spheroids h maximizing the drift for a given eccentricity of the spheroid, and align the rod along $\hat{y}$ direction so that the propeller has the maximum drift velocity along the $\hat{z}$ direction.

After straightforward calculations, we present our result for the maximum of v_E, the magnitude of which is $v_m = |\vec{v}_E|$:

$$\begin{aligned} v_E &= -\frac{1}{4}\frac{(X^A - Y^A)(X^C + Y^C + Y^H)k}{(X^A + Y^A)(X^C + Y^C) + 3X^AY^Ak^2}\,\dot\gamma\ell, \\ v_m &\equiv cV\ell = c\dot\gamma\ell \end{aligned} \tag{S14}$$

where $k \equiv h/\ell$, X's and Y's can be found in table 3.4 and 3.5 of Ref. [S1]. Depicted in Fig. S2 is the estimated c for prolate/oblate spheroids as a function of the shape parameter, i.e., the aspect ratio ($\equiv \ell/w$). When the aspect ratio becomes a large value, the prolate (oblate) spheroid resembles a rod (disk), and in this way, we are able to systematically evaluate c for the building blocks of various shapes. It is found that the estimated value of c remains of the order of $\mathcal{O}(10^{-2})$ or even smaller for all considered range of the aspect ratio, ℓ/w.

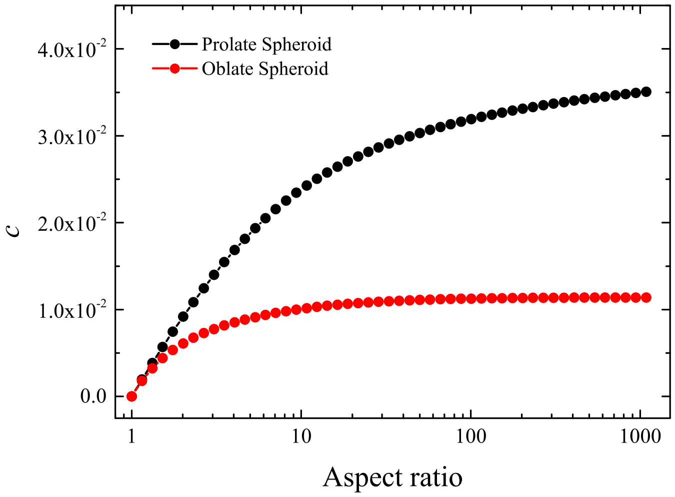

FIG. S2: c as function of aspect ratio, ℓ/w, for prolate (black) and oblate (red) spheroid. The aspect ratio are ranging from 1 to 10^3, and c gradually increases from zero and saturates to $\mathcal{O}(10^{-2})$.

III. LANGEVIN DYNAMICS SIMULATIONS

III. 1 Conversion equations for third-rank resistance tensors

In order to evaluate the mobility and resistance tensors, $\boldsymbol{\mu}$'s and $\boldsymbol{\zeta}$'s in Eq. (1), we decompose a rigid object of interest into an array of N spherical beads with radius a, and calculate the grand mobility tensors of the composite consisting of N beads. The resistance tensors associated with $\vec{U}$ or $\vec{\Omega}$ can then be evaluated from the grand mobility tensors through conversion equations, Eqs. (19) to (22) in Ref. [S5]. Here we derive the conversion equations for the third-rank tensors, $\boldsymbol{\zeta}^{\mathrm{te}}$ and $\boldsymbol{\zeta}^{\mathrm{re}}$, associated with the rate-of-strain field of $\mathbf{E}$, which were not given in Ref. [S5].

First, we introduce symbols for the grand resistance tensors, inverse of the grand mobility tensors, as $\left(\boldsymbol{\zeta}_{pq}^{mn}\right)_{ij}$. Superscripts m,n are either t or r, and p,q are bead indices ranging from 1 to N. Lastly, i,j,k are space indices and we use Einstein convention for summations involving i,j,k. Consider force $\vec{f}_p$ and torque $\vec{\tau}_p$ exerted on the p-th bead due to the rate-of-strain field $\mathbf{E}$,

$$\begin{aligned}\left(\vec{f}_p\right)_i &= \sum_{q=1}^{N}\left(\boldsymbol{\zeta}_{pq}^{\mathrm{tt}}\right)_{ij}(\mathbf{E})_{jk}\left(\vec{r}_q\right)_k \\ \left(\vec{\tau}_p\right)_i &= \epsilon_{ijk}\left(\vec{r}_p\right)_j\left(\vec{f}_p\right)_k+\sum_{q=1}^{N}\left(\boldsymbol{\zeta}_{pq}^{\mathrm{rt}}\right)_{ij}(\mathbf{E})_{jk}\left(\vec{r}_q\right)_k \ ,\end{aligned}$$

where $\vec{r}_q$ is the position of the q-th bead in relative to the center of mass of the considered object. Summing $\vec{f}_p$ and $\vec{\tau}_p$ over the beads, we obtain total force, $\vec{F}=\sum_{p=1}^{N}\vec{f}_p$, and total torque, $\vec{\mathcal{T}}=\sum_{p=1}^{N}\vec{\tau}_p$, acting on the object as

$$\begin{aligned}\vec{F}_i &= \sum_{p,q=1}^{N}\left(\boldsymbol{\zeta}_{pq}^{\mathrm{tt}}\right)_{ij}(\mathbf{E})_{jk}\left(\vec{r}_q\right)_k \\ \vec{\mathcal{T}}_i &= \sum_{p,q=1}^{N}\left[\varepsilon_{ijk}\left(\vec{r}_p\right)_j\left(\boldsymbol{\zeta}_{pq}^{\mathrm{tt}}\right)_{ki'}(\mathbf{E})_{i'j'}\left(\vec{r}_q\right)_{j'}\right] \ .\end{aligned}$$

These equations together with definitions $\vec{F}_i = \left(\boldsymbol{\zeta}^{\rm te}\right)_{ijk} (\mathbf{E})_{jk}$ and $\vec{T}_i = \left(\boldsymbol{\zeta}^{\rm re}\right)_{ijk} (\mathbf{E})_{jk}$, lead to

$$\left(\boldsymbol{\zeta}^{\rm te}\right)_{ijk} = \sum_{p,q=1}^{N} \left(\boldsymbol{\zeta}^{\rm tt}_{pq}\right)_{ij} (\vec{r}_q)_k \tag{S15}$$

$$\left(\boldsymbol{\zeta}^{\rm re}\right)_{ijk} = \sum_{p,q=1}^{N} \left[\varepsilon_{ij'k'} (\vec{r}_p)_{j'} \left(\boldsymbol{\zeta}^{\rm tt}_{pq}\right)_{k'j} (\vec{r}_q)_k\right] \tag{S16}$$

which are conversion relations used to obtain $\boldsymbol{\zeta}^{\rm te}$ and $\boldsymbol{\zeta}^{\rm re}$ from the grand mobility tensors.

III. 2 Discretized Langevin equations

In our study we perform Langevin dynamic simulations, adopting the conventional scheme (e.g, see Refs. [S5, S8, S9]). We first write the position Langevin equations, Eq. (1), that describe the dynamic evolutions of position $\vec{r}$ and orientation $\hat{\varphi}$ of a considered object as

$$\frac{dr_i}{dt} = U_i + \left(\mu^{\rm tt}_{ip}\zeta^{\rm te}_{pjk} + \mu^{\rm tr}_{ip}\zeta^{\rm re}_{pjk}\right) E_{jk} + \xi^{\rm t}_i\,, \tag{S17}$$

$$\frac{d\varphi_i}{dt} = \Omega_i + \left(\mu^{\rm rt}_{ip}\zeta^{\rm te}_{pjk} + \mu^{\rm rr}_{ip}\zeta^{\rm re}_{pjk}\right) E_{jk} + \xi^{\rm r}_i\,, \tag{S18}$$

where i, j, k are the coordinate indices, and Einstein summation convention is used. Here, μ^{mn} and ζ^{mn} are the mobility and resistance tensor, describing the coupling among translation (t), rotation (r), and a rate-of-strain field (e) components with $m, n = {\rm t, r, e}$ (see, e.g., Ch. 5.2 of Ref. [S1]). The vectorial random forces are correlated as $\langle \xi^\alpha_i(t)\xi^\beta_j(t')\rangle = 2k_BT\mu^{\alpha\beta}_{ij}\delta(t-t')$, according to the fluctuation-dissipation theorem. For simulations, we rescale all lengths by the bead radius a as $r = a\tilde{r}$, rescale all energies by the thermal energy, and make all relevant quantities into dimensionless forms with the tilde symbol:

$$\begin{aligned}
\mu^{\rm tt}_{ij} &= \frac{\tilde{\mu}^{\rm tt}_{ij}}{6\pi\eta Na},\quad \mu^{\rm tr}_{ij} = \frac{\tilde{\mu}^{\rm tr}_{ij}}{6\pi\eta Na^2},\quad \mu^{\rm rr}_{ij} = \frac{\tilde{\mu}^{\rm rr}_{ij}}{6\pi\eta Na^3},\\
\zeta^{\rm te}_{ijk} &= 6\pi\eta Na^2\tilde{\zeta}^{\rm te}_{ijk},\quad \zeta^{\rm re}_{ijk} = 6\pi\eta Na^3\tilde{\zeta}^{\rm re}_{ijk},\\
J_{ij} &= V\tilde{J}_{ij},\quad E_{ij} = V\tilde{E}_{ij},\quad \Omega_i = V\tilde{\Omega},\quad \varphi_i = \tilde{\varphi}_i,
\end{aligned}$$

where η is the viscosity of a fluid, N is the number of spherical beads constituting the object, k_B is the Boltzmann constant, T is temperature, and V is the flow field strength defined through the Jacobian matrix (e.g., see Eqs. (19) and (20) in the main text).

For numerical iterations, we discretize the Langevin equations with a time step Δ. The iterative Langevin equations in terms of the discrete time variable $p = t/\Delta$ then read

$$\begin{aligned}
\tilde{r}_i(p+1) &= \tilde{r}_i(p) + \tilde{\xi}^{\rm t}_i(p)\\
&+ \tilde{\mu}_0\left[\tilde{V}\tilde{J}_{ij}\tilde{r}_j + \tilde{V}\left(\tilde{\mu}^{\rm tt}_{ip}\tilde{\zeta}^{\rm te}_{pjk} + \tilde{\mu}^{\rm tr}_{ip}\tilde{\zeta}^{\rm re}_{pjk}\right)\tilde{E}_{jk}\right],
\end{aligned} \tag{S19}$$

$$\begin{aligned}
\tilde{\varphi}(p+1) &= \tilde{\varphi}(p) + \tilde{\xi}^{\rm r}_i(p)\\
&+ \tilde{\mu}_0\left[\tilde{V}\tilde{\Omega}_i + \tilde{V}\left(\tilde{\mu}^{\rm rt}_{ip}\tilde{\zeta}^{\rm te}_{pjk} + \tilde{\mu}^{\rm rr}_{ip}\tilde{\zeta}^{\rm re}_{pjk}\right)\tilde{E}_{jk}\right],
\end{aligned} \tag{S20}$$

where the dimensionless bare mobility, $\tilde{\mu}_0 = \Delta k_BT/6\pi\eta Na^3$, and the dimensionless flow field strength, $\tilde{V} = 6\pi\eta Na^3V/k_BT$, are introduced. They are chosen in our simulations as $\tilde{\mu}_0 = 10^{-3}$ or 10^{-4}, and $\tilde{V} = 30$, respectively. The variance of the rescaled random force is determined to satisfy the fluctuation-dissipation theorem, $\langle\tilde{\xi}^m_i(p)\tilde{\xi}^n_j(p')\rangle = 2\tilde{\mu}_0\tilde{\mu}^{mn}_{ij}\delta_{p,p'}$. Lastly, the mobility and resistance tensors (μ's and ζ's) are evaluated according to Eqs. (19)-(22) in Ref. [S5] and also Eqs. (S15) and (S16).

[S1] S. Kim and S. J. Karrila, *Microhydrodynamics: Principles and Selected Applications* (Butterworth-Heinemann, Boston, 1991).

[S2] M. Makino and M. Doi, J. Phys. Soc. Jpn, **73**, 2739 (2004).
[S3] J. G. Kirkwood and J. Riseman, J. Chem. Phys. **16** 512 (1948); J. Riseman and J. G. Kirkwood, J. Chem. Phys. **18**, 512 (1950).
[S4] V. A. Bloomfield, W. O. Dalton and K. E. Van Holde, Biopolymers **5**, 135 (1967); *ibid* **5**, 149 (1967).
[S5] B. Carrasco and J. Garcia de la Torre, J. Chem. Phys. **111**, 4817 (1999).
[S6] B. Carrasco and J. Garcia de la Torre, Biophys. J. **75**, 3044 (1999).
[S7] J. Garcia de la Torre and B. Carrasco, Biopolymers **63**, 163 (2002).
[S8] D. L. Ermak and J. A. McCammon, J. Chem. Phys. **69**, 1352-1360 (1978).
[S9] X. Sun, T. Lin, and J. D. Gezelter, J. Chem. Phys. **128**, 234107 (2008).
